# Health literacy in men and women with cardiovascular diseases and its association with the use of health care services - Results from the population-based GEDA2014/2015-EHIS survey in Germany

**DOI:** 10.1371/journal.pone.0208303

**Published:** 2018-12-06

**Authors:** Claudia Diederichs, Susanne Jordan, Olga Domanska, Hannelore Neuhauser

**Affiliations:** 1 Department of Epidemiology and Health Monitoring, Robert Koch Institute Berlin, Berlin, Germany; 2 German Center for Cardiovascular Research (DZHK), Partner Site Berlin, Germany; Sciensano, BELGIUM

## Abstract

**Background:**

Health literacy (HL), defined as the ability to access, understand, appraise and apply health information, offers a promising approach to reduce the development of cardiovascular diseases (CVD) and to improve the management of CVD in populations.

**Design:**

We used data from nationwide cross-sectional German Health Update (GEDA2014/2015-EHIS) survey. 13,577 adults ≥ 40 years completed a comprehensive standardized paper or online questionnaire including the short form of the European Health Literacy Survey Questionnaire (HLS-EU-Q16).

**Methods:**

We compared participants with and without CVD with regard to their HL. We also analyzed the association between HL level and health care outcomes among individuals with CVD, i.e. frequency of general practitioner or specialist consultations, hospitalization and treatment delay.

**Results:**

The percentage of “problematic” or “inadequate” HL, defined as “not sufficient” HL, was significantly higher in individuals with CVD compared to without CVD (men 41.8% vs. 33.6%, women 46.7% vs. 33.4%). Having CVD was independently associated with “not sufficient” HL after adjusting for age, education, income, health consciousness and social support (adjusted OR: men 1.36, women 1.64). Among participants with CVD, individuals with “inadequate” HL were more likely to have more than 6 general practitioner consultations (49.3% vs. 28.7%), hospitalization (46.6% vs. 36.0%) in the last 12 months and to experience delay in getting health care because of long waiting lists for an appointment (30.7% vs. 18.5%) compared to participants with “sufficient” HL.

**Conclusion:**

**“**Problematic” or “inadequate” HL is independently associated with CVD and health care use. This is a challenge and an opportunity for both CVD prevention and treatment.

## Introduction

The concept of health literacy (HL) was introduced for the first time in the 1970s [1]. Since then, many different definitions of health literacy have been developed and today, almost all have the same core elements including the skills to access, understand, appraise and apply health information [2–4]. Some newer definitions even go beyond this scope and consider the importance of HL in a broader context such as in disease prevention and health promotion [3]. Nutbeam [5] categorized HL skills as functional, interactive and critical health literacy. *Functional health literacy* refers to basic reading and writing skills. *Interactive health literacy* describes more advanced cognitive skills that enable individuals to derive meaningful information, to apply these in changing circumstances and engage in interactions. *Critical health literacy* refers to the most advanced skills which can be applied to critically analyze information and use these to exert greater control of life situations.

To measure health literacy in populations is challenging [4,6] and a variety of different instruments exist [7–10]. They can broadly be divided into performance-based tests, which assess the objective ability to read and understand health materials and subjective self-assessments. However, performance tests only capture the functional aspects of health literacy, whereas self-assessments ask for the self-perceived ability to access, understand, appraise and apply health information and also cover interactive and critical aspects of health literacy. Thus, they aim to measure the self-perceived capacity to function in the role of a patient within the health care system [3].

Based on this comprehensive understanding of HL, the European Health Literacy Project Consortium (HLS-EU) developed a questionnaire in 2011, the European Health Literacy Survey Questionnaire (HLS-EU-Q47), to measure and compare health literacy in eight European countries [11]. The study showed that “not sufficient” health literacy was not confined to a disadvantaged minority but that almost half of the European population (47.6%) had “inadequate” or “problematic” health literacy levels, ranging from 28.7% in the Netherlands to 62.1% in Bulgaria.

The implications of these findings were substantial, since low levels of health literacy are frequently associated with poorer disease management including limited risk factor and disease knowledge [12–14], lower use of preventive services [15], a reduced ability to take medications and to interpret labels and health messages properly [13,14] and lower adherence to medical treatment [16]. Especially people with cardiovascular diseases, which are characterized by their high preventive potential [17,18], and the importance of lifestyle changes and medication adherence in secondary prevention, can therefore profit from good health literacy levels [19].

Within the last few years, the concept of health literacy has become increasingly important and research has not only focused on the determinants and effects of health literacy, but the variety of recent studies range from an analysis of health literacy among caregivers of patients with heart failure in Italy [20], the relationship between health literacy and the use of health care services among refugees in Sweden [21] to the influence of health literacy on the acceptance of influenza and pertussis vaccinations in pregnant women in Spain [22].

In Germany, some studies have investigated the association between health literacy and chronic diseases in general [23] and myocardial infarction, stroke, hypertension and diabetes [24]. However, none of these studies has specifically focused on the association between HL and cardiovascular diseases and its influence on the use of health care services and unmet health care needs on the population level. Therefore, the aim of our analysis is to answer these pending research questions, based on data from nearly 15,000 individuals ≥ 40 years from the nationwide, population-based GEDA 2014/2015-EHIS survey.

## Materials and methods

### Study design and sample

This study is based on data from the cross-sectional “German Health Update 2014” (GEDA 2014/2015-EHIS), which is part of the nationwide health monitoring system administered by the Robert Koch Institute [25]. The GEDA 2014/2015-EHIS survey includes a wide range of health and sociodemographic questions based on self-reports of the participants and also questions of the European Health Interview Survey (EHIS) to provide comparable data about EU member states [26]. A two-stage, clustered sampling plan was used to select 301 communities, stratified by administrative districts and the BIK region size classes. Within selected communities, random samples of individuals ≥ 15 years with permanent residency in Germany were drawn from local population registries. Between November 2014 and June 2015, a total of 24,824 people either completed a paper or online questionnaire (mixed-mode-design). The response rate was 27.6%. More detailed information on the study design and sampling methods are described elsewhere [26,27].

Considering the low prevalence of cardiovascular disease in young individuals, we limited our analysis to an older population sample. Therefore, we excluded participants < 40 years (n = 8,092), with missing data on health literacy (n = 353) or cardiovascular diseases (n = 2,235), which left a total study sample of 14,144 participants ≥ 40 years (6,707 men and 7,437 women).

The study was approved by The Federal Commissioner for Data Protection and Freedom of Information, and written informed consent was obtained from all participants before the interview.

### Health literacy

Health literacy was assessed with the validated, short version (HLS-EU-Q16) of the European Health Literacy Survey Questionnaire [28]. The internal consistency of the HLS-EU-Q16 is reasonably high (the Cronbach´s alpha coefficient was 0.90 in a German sample of nearly 5000 adults [29] and 76.1% of the health literacy levels were consistent between the HLS-EU-Q47 and HLS-EU-Q16 [30].

The HLS-EU-Q16 consists of 16 it-ems reflecting the perceived difficulty to access, understand, appraise and apply health information in three different areas including health care, disease prevention and health promotion. Response options ranged from “very easy”, “fairly easy”, “fairly difficult” to “very difficult”. In order to calculate the overall health literacy score, the responses were dichotomized and “very easy” and “easy” received 1 point and “difficult” and “very difficult” 0 points. For all participants who had answered at least 14 out of 16 questions, the points were added up to reflect the overall health literacy score, categorized as “sufficient” (13–16 points), “problematic” (9–12 points) or “inadequate” (0–8 points) HL. If responses to more than two questions were missing, the overall score was set to missing [30]. The categories “problematic” and “inadequate” were combined for logistic regression analysis and defined as “not sufficient” health literacy.

### Other variables

Sociodemographic variables included sex, age (40–49 years / 50–59 years / 60–69 years / 70–79 years / ≥ 80 years) education (high / medium / low) and monthly net equivalent income (> 4000 € / 3000–3999 € / 2000–2999€ / < 2000 €). Health consciousness, defined as “the degree to which someone attends to or focuses on his or her health, an inner state of self-attention to self-relevant cues reflected in both thought and somatic feeling “[31], is positively associated with health literacy [32]. It was assessed with the question “In general, how much do you take care of your health?” on a five-point Likert scale from “a lot”, to “not at all” as answer options [31].

The Oslo 3-Items Social Support Scale was used to measure the level of perceived social support, categorized as “poor” (3–8 points), “moderate” (9–11 points) and “strong” (12–14 points) [33]. Questions concerning the use of health care services were dichotomized (yes/no) and assessed whether the participants had more than 6 general practitioner (GP) consultations, more than 6 specialist consultations or were hospitalized, all referring to a time period of the last 12 months. Two questions assessed whether the participants had experienced delay in getting health care within the last 12 month because of long waiting lists or because of distance or transport problems [34]. The answer option “no need for health care” was set to missing. Unmet health care needs are defined as the difference between medical services judged necessary to deal appropriately with health problems and services actually received [35]. As part of the standardized European Health Interview Survey (EHIS) the reasons for unmet health care needs are an important indicator to assess equity to health care services [36].

Participants with at least one of the following diseases including myocardial infarction, stroke, heart failure or coronary heart disease either present in the last 12 months or diagnosed by a physician any time during their life course were classified as participants with cardiovascular diseases (CVD).

### Statistical analyses

**Fig 1:**
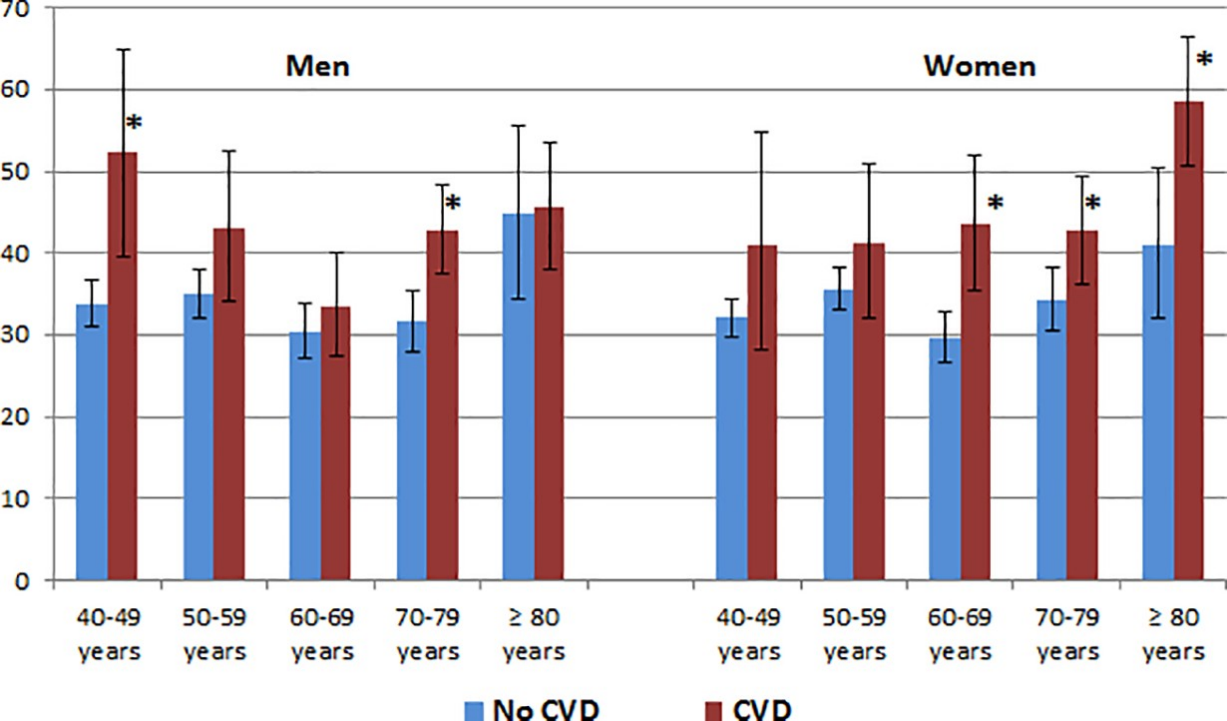
Health literacy and cardiovascular diseases. Percentage of the population with “problematic” or “inadequate” health literacy according to cardiovascular health status.

All analyses were performed stratified for men and women. We showed the distribution of the study population according to selected sociodemographic variables given as percentages and p-values were calculated with the Chi-Square-Test (Table 1). In order to compare individuals without and with CVD, we calculated the percentage with answer categories “fairly difficult”or “very difficult” for each item of the HLS-EU-Q16 (Tables 2 and 3) and the percentage with “problematic” or “inadequate” (0–12 points) versus “sufficient” health literacy (13–16 points) (Table 4 and Fig 1). In individuals with CVD, we analyzed the association between health literacy and selected health care outcomes.

**Table 1:** Overview on the study population of GEDA 2014/2015-EHIS participants ≥ 40 years with complete information on health literacy stratified for men and women (n = 14,144, unweighted).

		MEN			WOMEN		
		No CVD (n = 5,352)	CVD^1^ (n = 1,355)	p-value	No CVD (n = 6,495)	CVD^1^ (n = 942)	p-value
		%	%		%	%	
**Age**	40–49 years	33.2	7.8	<0.001	29.6	6.7	<0.001
	50–59 years	32.4	17.8		30.1	11.5	
	60–69 years	18.5	24.6		19.6	18.3	
	70–79 years	13.0	33.3		16.0	37.2	
	≥ 80 years	2.9	16.5		4.7	25.8	
**Education**	High	33.3	29.4	<0.001	18.2	9.7	<0.001
	Medium	56.7	55.7		61.3	49.2	
	Low	10.0	14.9		20.5	41.2	
**Equivalised income (imputed)**	> 4000 €	6.8	3.8	<0.001	3.7	2.0	<0.001
	3000–3999€	8.4	5.6		6.8	3.5	
	2000–2999 €	23.0	16.7		19.0	12.4	
	< 2000€	61.8	74.0		70.6	82.2	
**Health consciousness**	Very high / high	45.0	53.0	0.001	56.0	60.6	0.054
	Medium	47.8	40.1		39.2	34.5	
	Low / None	7.2	6.9		4.8	4.9	
**Social support (Oslo-Social Support Scale)**	Strong	26.3	23.3	0.018	30.9	24.0	0.002
	Medium	56.0	55.4		52.4	53.8	
	Poor	17.7	21.4		16.8	22.2	
**> 6 GP consultations in the last 12 month**	Yes	10.0	33.2	<0.001	13.0	35.1	<0.001
**> 6 specialist consultations in the last 12 months**	Yes	7.1	15.2	<0.001	9.9	16.8	<0.001
**Hospitalized in the last 12 months**	Yes	14.1	38.0	<0.001	14.9	38.1	<0.001
**Delay in getting health care because of long waiting lists**	Yes	20.7	23.0	0.145	24.3	22.6	0.357
**Delay in getting health care due to distance or transport problems**	Yes	2.4	5.5	0.001	3.9	8.9	<0.001
**Health literacy (HLS-EU-Q16)**	Inadequate (0–8)	9.5	13.9	<0.001	9.2	18.3	<0.001
	Problematic (9–12)	24.1	27.9		24.2	28.4	
	Sufficient (13–16)	66.4	58.2		66.6	53.3	

1) Participants with at least one of the following diseases: myocardial infarction, stroke, heart insufficiency, coronary heart disease either in the last 12month or diagnosed by a physician any time during their life course. All results are weighed to adjust the sample to the German standard population from 31. December 2011; GP = general practitioner

**Table 2:** Association of cardiovascular diseases and difficulties in single health literacy items (HLS-EU-Q16) in men (n = 6,707, unweighted).

Q16	*On a scale from very easy to very difficult*. *how easy would you say it is to* …	Men (%) with answer categories „very / fairly difficult“		Model 1^2^	Model 2^3^
		No CVD	CVD^1^	age adjusted OR	multivariate adjusted OR
	**Access / obtain health relevant information**				
8	*…find information on how to manage mental health problems like stress or depression*?	29.0	34.2	1.30*	1.21*
13	*…find out about activities that are good for your mental well-being*?	19.8	26.8	1.60**	1.46**
2	*…find out where to get professional help when you are ill*?	10.0	12.0	1.34*	1.23
1	*…find information on treatments of illnesses that concern you*?	14.0	18.7	1.28*	1.15
	**Understand health relevant information**				
15	*…understand information in the media on how to get healthier*?	20.9	31.7	1.53**	1.40**
14	*…understand advice on health from family members or friends*?	12.4	16.9	1.45*	1.30*
3	*…understand what your doctor says to you*?	12.2	15.9	1.47*	1.32*
10	*…understand why you need health screenings*?	5.6	3.4	0.74	0.65
4	*…understand your doctor’s or pharmacist’s instruction on how to take a prescribed medicine*?	3.0	5.9	1.70*	1.59*
9	*…understand health warnings about behavior such as smoking*, *low physical activity and drinking too much*?	4.2	6.7	2.18**	1.90*
	**Appraise / judge health relevant information**				
11	*…judge if the information on health risks in the media is reliable*?	43.2	45.8	1.11	1.05
5	*…judge when you may need to get a second opinion from another doctor*?	31.3	34.3	1.18	1.10
16	*…judge which everyday behavior is related to your health*?	10.9	13.9	1.49*	1.32*
	**Apply / use health relevant information**				
12	*…decide how you can protect yourself from illness based on information in the media*?	36.7	42.7	1.25*	1.20*
6	*…use information the doctor gives you to make decisions about your illness*?	19.0	22.7	1.36*	1.23
7	*…follow instructions from your doctor or pharmacist*?	4.9	6.4	1.49*	1.38*

1) Participants with at least one of the following diseases: myocardial infarction, stroke, heart insufficiency, coronary heart disease either in the last 12 month or diagnosed by a physician any time during their life course, persons without cardiovascular diseases are the reference category

2) adjusted for age

3) adjusted for age, education, income, health consciousness, social support

*p < 0.05

**p < 0.001

**Table 3:** Association of cardiovascular diseases and difficulties in single health literacy items (HLS-EU-Q16) in women (n = 7,437, unweighted).

Q16	*On a scale from very easy to very difficult*. *how easy would you say it is to* …	Women (%) with answer categories „very / fairly difficult“		Model 1^2^	Model 2^3^
		No CVD	CVD^1^	age adjusted OR	multivariate adjusted OR
	**Access / obtain health relevant information**				
8	*…find information on how to manage mental health problems like stress or depression*?	32.5	36.7	1.18	1.11
13	*…find out about activities that are good for your mental well-being*?	16.0	26.7	1.52**	1.43*
2	*…find out where to get professional help when you are ill*?	12.4	17.8	1.64**	1.47*
1	*…find information on treatments of illnesses that concern you*?	17.7	31.1	1.75**	1.63**
	**Understand health relevant information**				
15	*…understand information in the media on how to get healthier*?	19.7	30.2	1.37*	1.32*
14	*…understand advice on health from family members or friends*?	11.3	17.3	1.33*	1.15
3	*…understand what your doctor says to you*?	13.3	17.4	1.25	1.11
10	*…understand why you need health screenings*?	3.5	8.1	1.77*	1.60*
4	*…understand your doctor’s or pharmacist’s instruction on how to take a prescribed medicine*?	2.9	9.7	2.09**	1.82*
9	*…understand health warnings about behavior such as smoking*, *low physical activity and drinking too much*?	3.1	8.7	2.39**	2.18**
	**Appraise / judge health relevant information**				
11	*…judge if the information on health risks in the media is reliable*?	43.7	48.6	1.15	1.17
5	*…judge when you may need to get a second opinion from another doctor*?	32.9	39.3	1.25*	1.17
16	*…judge which everyday behavior is related to your health*?	9.3	19.4	2.15**	1.95**
	**Apply / use health relevant information**				
12	*…decide how you can protect yourself from illness based on information in the media*?	35.1	41.3	1.14	1.11
6	*…use information the doctor gives you to make decisions about your illness*?	22.9	29.5	1.32*	1.23*
7	*…follow instructions from your doctor or pharmacist*?	4.9	10.6	1.87**	1.71*

1) Participants with at least one of the following diseases: myocardial infarction, stroke, heart insufficiency, coronary heart disease either in the last 12 month or diagnosed by a physician any time during their life course, persons without cardiovascular diseases are the reference category

2) adjusted for age

3) adjusted for age, education, income, health consciousness, social support

*p < 0.05

**p < 0.001

**Table 4:** Association of cardiovascular diseases^1^ and “problematic” or “inadequate” health literacy levels (HLS-EU-Q16) stratified for sex and age groups (n = 14,144, unweighted) with OR and 95%-CI.

		40–49 years			50–59 years			60–69 years			70–79 years			≥ 80 years			Overall		
		% with proble-matic / inade-quate HL	Model 1^2^	Model 2^3^	% with proble-matic / inade-quate HL	Model 1^2^	Model 2^3^	% with proble-matic / inade-quate HL	Model 1^2^	Model 2^3^	% with proble-matic / inade-quate HL	Model 1^2^	Model 2^3^	% with proble-matic / inade-quate HL	Model 1^2^	Model 2^3^	% with proble-matic / inade-quate HL	Model 1^4^	Model 2^5^
			OR	OR		OR	OR		OR	OR		OR	OR		OR	OR		OR	OR
**Men**	**No CVD**	**33.8**	1.00	1.00	**35.0**	1.00	1.00	**30.4**	1.00	1.00	**31.6**	1.00	1.00	**44.8**	1.00	1.00	**33.6**	1.00	1.00
		31.1–36.7	-	-	32.1–38.1	-	-	27.1–34.0	-	-	27.9–35.5	-	-	34.3–55.7	-	-	32.0–35.2	-	-
	**CVD**	**52.3**	2.15*	1.84*	**43.0**	1.40	1.33	**33.5**	1.15	1.02	**42.9**	1.62*	1.52*	**45.6**	1.04	1.02	**41.8**	1.41**	1.36**
		39.5–64.9	1.27–3.63	1.05–3.24	34.1–52.5	0.93–2.12	0.87–2.05	27.4–40.1	0.83–1.60	0.73–1.44	37.5–48.4	1.21–2.18	1.12–2.07	37.9–53.6	0.61–1.76	0.58–1.77	38.4–45.2	1.19–1.67	1.16–1.60
**Women**	**No CVD**	**32.1**	1.00	1.00	**35.5**	1.00	1.00	**29.7**	1.00	1.00	**34.2**	1.00	1.00	**40.9**	1.00	1.00	**33.4**	1.00	1.00
		29.8–34.5	-	-	33.0–38.2	-	-	26.6–32.9	-	-	30.5–38.4	-	-	32.0–50.4	-	-	32.0–34.8	-	-
	**CVD**	**40.9**	1.46	1.47	**41.3**	1.28	1.01	**43.5**	1.83*	1.59*	**42.7**	1.43*	1.52*	**58.7**	2.06*	2.02*	**46.7**	1.61**	1.64**
		28.3–54.8	0.83–2.57	0.82–2.61	32.2–51.0	0.84–1.93	0.66–1.55	35.5–51.9	1.28–2.61	1.09–2.34	36.2–49.5	1.03–1.97	1.08–2.14	50.6–66.4	1.23–3.45	1.17–3.50	42.6–50.9	1.33–1.94	1.36–1.98

1) Participants with at least one of the following diseases: myocardial infarction, stroke, heart insufficiency, coronary heart disease either in the last 12 month or diagnosed by a physician any time during their life course, persons without cardiovascular diseases are the reference category

2) unadjusted

3) adjusted for education, income, health consciousness, social support

4) adjusted for age

5) adjusted for age, education, income, health consciousness, social support

*p < 0.05

**p < 0.001

On average, health literacy decreases with age and participants with CVD are older than those without CVD. Therefore we reported age-adjusted ORs (Model 1) from logistic regression analyses. In a second step, models were further adjusted for education, income, health consciousness and social support (Model 2) [30]. We presented results stratified by gender because women were significantly older (60.1 years, 95%-CI: 59.7–60.4) than men (58.6 years, 95%-CI: 58.3–58.9) and we found significant interactions between sex and age for some single items of the HLS-EU-Q16. Not at least, the opportunity to present results stratified for sex and partly for age groups was one of the major benefits of our large study sample.

All analyses were performed using STATA SE14 (StataCorp LP, Texas, US). Weights and survey commands were used to adjust the sample to the German standard population from 31. December 2011 with respect to age, sex, level of education and regional distribution of the population [27].

## Results

Table 1 shows the distribution of selected socio-demographic characteristics and health care use variables stratified for individuals with and without cardiovascular diseases. Overall, CVD including myocardial infarction, stroke, heart failure or coronary heart disease were reported by 20.2% (n = 1,355) of the male and 12,6% (n = 942) of the female population ≥40 years.

The percentage of individuals with self-reported difficulties (answer categories “fairly difficult” or “very difficult”) for each item of HLS-EU-Q16 ranged between 2.9% and 48.6% (Tables 2 and 3). Individuals tended to report more difficulties in items assessing the capacity to judge or apply complex health information as well as in items concerning their mental well-being, in contrast to questions assessing the ability to access or understand more simple health information. In detail, men and women had the most difficulties “to judge if the information on health risks in the media is reliable?” (43.2% - 48.6% reported difficulties, depending on sex and CVD status) followed by “to decide how you can protect yourself from illness based on information in the media?” (35.1% - 42.7%) and “to judge when you may need to get a second opinion from another doctor?” (31.3% - 39.3%). The items with the fewest difficulties were “to understand your doctor’s or pharmacist’s instruction on how to take a prescribed medicine?” (2.9% - 9.7%) and “to understand health warnings about behavior such as smoking, low physical activity and drinking too much?” (3.1% - 8.7%).

Differences between individuals with and without cardiovascular diseases were more pronounced in women compared to men (Tables 2 and 3). Regarding absolute differences, the percentage of men with CVD who reported difficulties were between 1.5 and 10.8 percentage points higher compared to men without CVD, depending on the item on the HLS-EU-Q16. In women, these differences were slightly larger and ranged between 4.1 and 13.4 percentage points. More precisely, the greatest difference in men was observed for the item “to understand information in the media on how to get healthier?” (31.7% with CVD versus 20.9% without CVD) and in women for the item “to find information on treatments of illnesses that concern you?” (31.1% versus 17.7%). In age-adjusted logistic regression analyses, CVD was associated with difficulties in 13 out of 16 HL items in men and in 12 out of 16 items in women, with ORs ranging from 1.28 to 2.18 in men and from 1.25 to 2.39 in women. After further adjustment for education, income, health consciousness and social support, the associations became slightly weaker and for a few items they were no longer statistically significant.

By combining the items to an overall health literacy score (Table 4 and Fig 1), 33.6% of men without CVD and 41.8% of men with CVD had “problematic” or “inadequate” HL (0–12 points). In women, the respective numbers were 33.4% versus 46.7%. Having CVD was significantly associated with “problematic” or “inadequate” HL in men and in women both after adjusting for age alone (OR 1.41 in men and 1.61 in women) as well as after further multivariate adjustment (OR 1.36 in men and 1.64 in women). Stratification for age groups revealed that in men the greatest absolute differences in health literacy levels between individuals with and without CVD were found in 40–49 year old men (18.5% points) while in women, this age-related observation was reversed and the greatest absolute difference was found in women ≥80 years (17.8% points).

In individuals with CVD, health literacy was clearly associated with the use of several health services (Table 5) in age-adjusted and multivariate-adjusted comparisons. 49.3% of participants with “inadequate” levels of health literacy had more than 6 general practitioner consultations in the last 12 months, compared to 28.7% with “sufficient” health literacy. Individuals with “inadequate” health literacy were hospitalized more frequently (46.6% versus 36.0%) in the last year compared to participants with “sufficient” health literacy. Furthermore, unmet health care needs, such as delay in getting health care because of long waiting lists (30.7% versus 18.5%) or transport problems (16.3% versus 3.2%) were significantly higher in men and women with “inadequate” HL compared to individuals with “sufficient” HL.

**Table 5:** Association of health literacy levels (HLS-EU-Q16) and health care use in participants with cardiovascular diseases^1^ > = 40 years.

Health literacy level	% of participants with CVD		Model 1^2^		Model 2^3^	
	%	(95%-CI)	OR	(95%-CI)	OR	(95%-CI)
	**> 6 GP consultations in the last 12 months (n = 2,198)**					
**Sufficient**	28.7	25.6–32.1	1.00	-	1.00	-
**Problematic**	35.9	31.2–40.8	1.37*	1.03–1.83	1.41*	1.05–1.90
**Inadequate**	49.3	42.5–56.1	2.32**	1.71–3.16	2.40**	1.75–3.29
	**> 6 specialist consultations in the last 12 months (n = 2,127)**					
**Sufficient**	14.6	12.5–17.1	1.00	-	1.00	-
**Problematic**	16.3	13.2–20.1	1.14	0.83–1.55	1.20	0.86–1.66
**Inadequate**	19.6	14.9–25.2	1.39	0.96–2.03	1.67*	1.10–2.51
	**Hospitalized in the last 12 months (n = 2,278)**					
**Sufficient**	36.0	32.8–39.4	1.00	-	1.00	-
**Problematic**	37.2	33.1–41.5	1.04	0.82–1.32	1.04	0.81–1.34
**Inadequate**	46.6	40.1–53.3	1.50*	1.13–2.00	1.51*	1.11–2.04
	**Delay in getting health care because of long waiting lists (n = 2,164)**					
**Sufficient**	18.5	15.8–21.5	1.00	-	1.00	-
**Problematic**	27.2	23.0–31.7	1.75**	1.30–2.34	1.64*	1.23–2.10
**Inadequate**	30.7	24.8–37.3	2.21**	1.54–3.15	2.08**	1.43–3.01
	**Delay in getting health care due to distance or transport problems (n = 2,163)**					
**Sufficient**	3.2	2.3–4.5	1.00	-	1.00	-
**Problematic**	9.3	6.4–13.2	3.03**	1.76–5.22	2.93*	1.76–5.22
**Inadequate**	16.3	11.7–22.2	5.59**	3.29–9.51	5.63**	3.29–9.51

1) Participants with at least one of the following diseases: myocardial infarction, stroke, heart insufficiency, coronary heart disease either in the last 12 month or diagnosed by a physician any time during their life course, persons without cardiovascular diseases are the reference category

2) adjusted for age, sex

3) adjusted for age, sex, education, income, health consciousness, social support

*p < 0.05

**p < 0.001

## Discussion

Our analysis showed that 41.8% of the male and 46.7% of the female population ≥ 40 years with cardiovascular diseases in Germany reported difficulties in accessing, understanding, appraising or applying health relevant information in their daily life. Low health literacy levels were particularly common in individuals with CVD. The association between CVD and “not sufficient” HL was independent of age, education, income, health consciousness and social support. Furthermore, “inadequate” health literacy was associated with increased general practitioner and specialist consultations, more frequent hospitalizations and unmet health care needs such as delay in getting health care.

In general, the diversity of instruments which measure different aspects of health literacy [7,8,10] makes it difficult to compare population estimates of health literacy levels [6]. For example the prevalence of low health literacy in an Australian population varied between 6.8% and 26.0%, depending on the instruments used [37]. Therefore, for reasons of comparability we will primarily discuss studies that used the long or short form of the HLS-EU. Still, population-based estimates of the number of people with “problematic” or “inadequate” health literacy levels in Germany differed considerably with a range between 22.9% and 66.4% [23,24,29,38,39] These variations can partly be explained by methodological differences in the calculation of HL levels, for example the HLS-EU-Q47 uses four instead of three HL categories and in some studies the HLS-EU-Q16 score was transformed accordingly [24,38] Furthermore, the age and socio-demographic structure of the population samples were very diverse, for example some studies analyzed the whole population ≥ 15 years [38] whereas others exclusively looked at 45 to 83 year old individuals [24] or at young people (15 to 25 years) with low education levels [39]. However, in our analysis, 33.6% of men without CVD and 41.8% of men with CVD had “problematic” or “inadequate” health literacy. In women, the respective numbers were 33.4% versus 46.7% and thus our results fit well into the spectrum outlined above.

Moreover, in line with our findings, several German studies using the HLS-EU-Q16 consistently reported the highest percentage of difficulties for the question “to judge if the information on health risks in the media is reliable?” [21,30,34]. Interestingly, the other two questions in the HLS-EU-Q16 instrument concerning the perception of health information in the media, such as “to decide how you can protect yourself from illness based on information in the media?” and “to understand information in the media on how to get healthier?” received an equally high percentage of responders with problems in the German literature [24,29,40] and our analysis. At the same time, the least difficulties were commonly reported for items evaluating communication and interactions between patients and physicians or pharmacists, such as “to understand your doctor’s or pharmacist’s instruction on how to take a prescribed medicine?” or “to follow instructions from your doctor or pharmacist?” [24,29,40]. These results suggest that on the one hand, there are uncertainties or a “healthy” distrust on the reliability of health information derived from newspapers, magazines, television and the internet. On the other hand, despite the increasing importance of the internet for medical information [41], that health care professionals remain to be most trusted contact persons for health related concerns. However, apart from simple instructions, a considerable percentage of the population had difficulties in more complex interactions with the health care system, such as “to use information the doctor gives you to make decisions about your illness?” or “to judge when you may need to get a second opinion from another doctor?” [24,29,40] These findings indicate that there is still a high potential for improving the doctor-patient communication and more explicitly, for health care professionals to interact in a clear and understandable way and to actively integrate patients in decision making processes.

Our analysis showed that consistently in both sexes, the largest relative differences between individuals with and without CVD were observed for the item “to understand health warnings about behavior such as smoking, low physical activity and drinking too much?” and in women for “to judge which everyday behavior is related to your health?”. Although from a public health perspective, the absolute number of individuals who had difficulties in these specific HL items was rather low, these two questions had the strongest link to the prevention and management of cardiovascular diseases. For health care professionals, these results emphasize not only the importance to actively inform and support CVD patients regarding the positive effects of lifestyle changes and medication adherence in primary and secondary prevention, but to stress the patients`influence and responsibility for their own health.

Our study further highlighted that individuals with CVD reported significantly more difficulties in items related to mental health issues, such as “to find out about activities that are good for your mental well-being” for both sexes and “to find information on how to manage mental health problems like stress or depression?” for men. Considering that CVD patients have an increased risk for depression [42], these findings support the need to actively provide individual assistance to deal with mental health issues during the treatment process.

We found that “not sufficient” HL in individuals with CVD was associated with more frequent general practitioner and specialist visits. Two other German studies observed a similar association for both sexes [38] or only in men [24]. The literature shows that individuals without “sufficient” HL are more likely to experience adverse health outcomes such as poorer overall health status [43–45], lower patient satisfaction [46], mortality [15,47,48] and higher health care costs [46, 49,50]. However, the majority of studies used instruments which captured only the functional aspects of health literacy and the results are difficult to compare with our analysis, which also measured interactive and critical HL.

Our finding, that “not sufficient” HL was related to increased hospitalization were confirmed by the majority of studies [15,51,52], but they all used performance-based instruments. In contrast to this, two analyses in heart failure patients in the US [47] and hospitals patients in Australia [53] which were also based on subjective instruments similar to the HLS-EU-Q16, did not find an association between low HL and increased hospitalization. However, these inconsistent results are not surprising in view of the large differences between health care systems. Therefore, further research is required to investigate the underlying mechanisms that influence the relationship between health literacy and the use of health care services in different countries.

To our knowledge, this is the first study in Germany that shows that individuals with CVD and without “sufficient” health literacy level were more likely to have delay in getting health care services because of long waiting lists or because of distance or transportation problems. In line with our findings, low health literacy was associated with higher unmet information needs in German breast cancer patients [54] and with delay in needed care and difficulty finding a provider in an American population sample [55]. Here, further studies could help to identify the barriers which exactly prevent people without “sufficient” HL to access the health care system in Germany.

One of the major strengths of this study are the national survey design based on a general population sample and the large study sample with more than 14,144 respondents who completed the HLS-EU-Q16. Thus, we were able to analyze health literacy levels in subgroups of the population and we chose individuals with cardiovascular diseases as the number one cause of death worldwide [56]. Furthermore, weights were applied to adjust the sample to the German standard population which increased the representativeness of the results. However, response rates were moderate and although this has become a common problem in epidemiological studies [57], this might reduce the generalizability of our findings. In addition to this, the GEDA2014/15-EHIS survey was restricted to participants with German language skills and therefore health literacy levels in the population are probably overestimated. Selection bias may further increase this overestimation, since people with low reading ability, which is closely related to the functional aspects of health literacy, are less likely to participate in online and paper questionnaires.

One inherent weakness of all subjective health literacy instruments is that they are not able to distinguish between fundamentally different reasons why people report difficulties in dealing with the health care system [58]. For example, well-educated, intelligent individuals might be more aware of their own limitations in their role as patients and the complexity of the system, whereas a low locus of control or bad experiences might also cause people to report problems. Furthermore, our analysis and the majority of previously discussed studies use cross-sectional designs, which make it impossible to analyze causal relationships and the direction of effects. Although health literacy is primarily understood as an impact factor on several health outcomes [15], the self-perceived health literacy is vice versa influenced by confidence, social resources and individual skills [7]. This has to be kept in mind when interpreting the results.

## Conclusion

Health literacy in individuals with CVD is a rather new area of research and it provides the opportunity to identify specific problems in this population subgroup, for example more self-reported difficulties to deal with mental health issues and reduced knowledge of CVD risk factors. In order to provide a more comprehensive analysis on the complex prevention, treatment and rehabilitation aspects in CVD, the development of a disease-specific instrument may be the next useful step. Other HL instruments that were developed for example for cancer [59] and diabetes [60], can serve as appropriate models.

Nutbeam [4] calls for improving health literacy through the implementation of national strategies, the provision of information, effective communication and structured education. Although actions to increase health literacy do not always translate into improved preventive behavior, disease management or health outcomes, some interventions have shown success and seem worth pursuing [16,61,62]. Several HL interventions for patients with cardiovascular risk factors and diseases were positively evaluated, for example tailored feedback over a period of 6 month significantly improved the ability to manage hypertension in hypertensive veterans [63], nurse-coordinated care reduced the 10-year mortality risk in patients with coronary artery disease [18] and better knowledge of risk factors in coronary heart disease patients was linked to improved adherence to lifestyle changes and medication [64]. How well patients understand and manage the disease is not only influenced by their own health literacy level but also by the communicative skills of their physicians. Therefore, one important aspect is that health care professionals know how to identify patients without “sufficient” health literacy and to decide whether individual support and education is advisable. Some indications of lower HL are patient behaviors such as postponing decision making, non-compliance with recommended treatment, taking a companion to the appointment or making excuses such as “I forgot my glasses”[65]. And beyond the individual doctor-patient relationship, structural efforts are needed to improve the health literacy in the general population.

And finally, long term adherence to lifestyle changes and medication, even in individuals who have already experienced cardiovascular diseases, is still unsatisfactory [66], especially in population groups with a low socio-economic status [67,68]. A low SES is in turn closely associated with “not sufficient” levels of health literacy, for example in a German population sample, “problematic” or “inadequate” HL was prevalent in 78.8% with a low compared to 37.8% with a high SES [23]. National strategies and interventions to increase HL have therefore been regarded as a promising pathway not only to improve problematic disease management and adverse health outcomes, but in the long-term, to reduce the existing gap between different SES groups in terms of CVD disease prevalence and mortality [16,69].
